# The ratio of alanine aminotransferase to high-density lipoprotein cholesterol is positively correlated with the insulin resistance in American adults: a population-based cohort study

**DOI:** 10.3389/fmed.2024.1418364

**Published:** 2024-06-19

**Authors:** Xinhe Zhou, Jing Xu, Huifang Dai

**Affiliations:** Department of Endocrinology, The Second Affiliated Hospital and Yuying Children’s Hospital of Wenzhou Medical University, Wenzhou, China

**Keywords:** obesity, race, alanine aminotransferase, insulin resistance, diabetes

## Abstract

**Introduction:**

Previous studies have demonstrated a correlation between the ratio of alanine aminotransferase to high-density lipoprotein cholesterol (ALT/HDL-C) in the serum and the risk of diabetes. However, no existing study has investigated the association between insulin resistance (IR) and ALT/HDL-C. Therefore, this study aims to explore the association between ALT/HDL-C and IR in American adults.

**Methods:**

A total of 7,599 adults selected from the National Health and Nutrition Examination Survey (NHANES) in 2013 to 2020 were studied. IR was assessed based on the homeostatic model assessment of insulin resistance (HOMA-IR). And the association between IR and ALT/HDL-C was assessed through multiple logistic regression, generalized smooth curve fitting and subgroup analyses.

**Results:**

Multiple logistic regression analysis indicated a significant correlation between IR and ALT/HDL-C, with odds ratios (OR) of 1.04 (95% CI = 1.02–1.05) in males and 1.04 (95% CI = 1.02–1.07) in females. A non-linear association and saturation effect between ALT/HDL-C and IR risk were identified, with an inverted L shaped curve and an inflection point at 33.62. The area under the ROC curve (AUC) of ALT/HDL-C was significantly larger (AUC = 0.725 for males and 0.696 for females, all *p* < 0.01) compared with the use of ALT, HDL-C, AST and AST/ALT. Subgroup analysis showed a significantly higher independent association in obese individuals and individuals aged ≥50 years (All *P* interaction <0.05).

**Conclusion:**

Elevated ALT/HDL-C demonstrates a significant correlation with IR, which can be used as a potential indicator of IR in American adults.

## Introduction

IR is widely recognized as a significant contributing factor in various pathological conditions, including diabetes, atherosclerosis, hypertension and metabolic syndrome (MetS). Therefore, the accurate measurement on IR is of utmost importance. The hyperinsulinemic-euglycemic clamp is considered as the gold standard for IR. However, its routine clinical application is hindered by issues related to replicability, cost, accessibility and reproducibility (1–5). As an alternative, HOMA-IR is considered as an index widely used in adults (6). Although HOMA-IR is commonly adopted in adults, its reliance on fasting plasma insulin measurements poses challenges within clinical settings. Consequently, there is a demand for a diagnostic test with accuracy, cost-effectiveness and simplicity in predicting IR.

ALT is commonly considered as an epidemiological marker for NAFLD, which is associated with an increased risk of developing diabetes (7). Furthermore, there is evidence suggesting that elevated ALT levels are linked to hepatic IR, potentially contributing to the onset of diabetes (8). Additionally, decreased HDL-C levels have been implicated in the pathogenesis of IR and MetS (9–13). Recently, Cao et al. conducted a study investigating the combination of HDL-C and ALT, and the findings suggest that the ALT/HDL-C ratio serves as a valuable novel predictor for the risk in the development of diabetes in Japanese (14). He et al. conducted a study on a total of 116,251 Chinese people and found a positive correlation between the risk in the development of diabetes and the ALT/HDL-C (15). However, the existence of an association between IR risk and ALT/HDL-C remains unclear. In order to explore this hypothesis, the current study sought to analyze the correlation between ALT/HDL-C and IR through a substantial sample of American adults derived from NHANES.

## Methods

### Research subjects

The data analyzed in this study were obtained from NHANES (2013–2020), with a stratified, multi-stage probability and complex sample of the uninstituted population in America. The cross-sectional surveys were conducted by NCHS. Further information regarding NHANES methods can be accessed at www.cdc.gov/nchs/NHANEs/.

**Figure 1:**
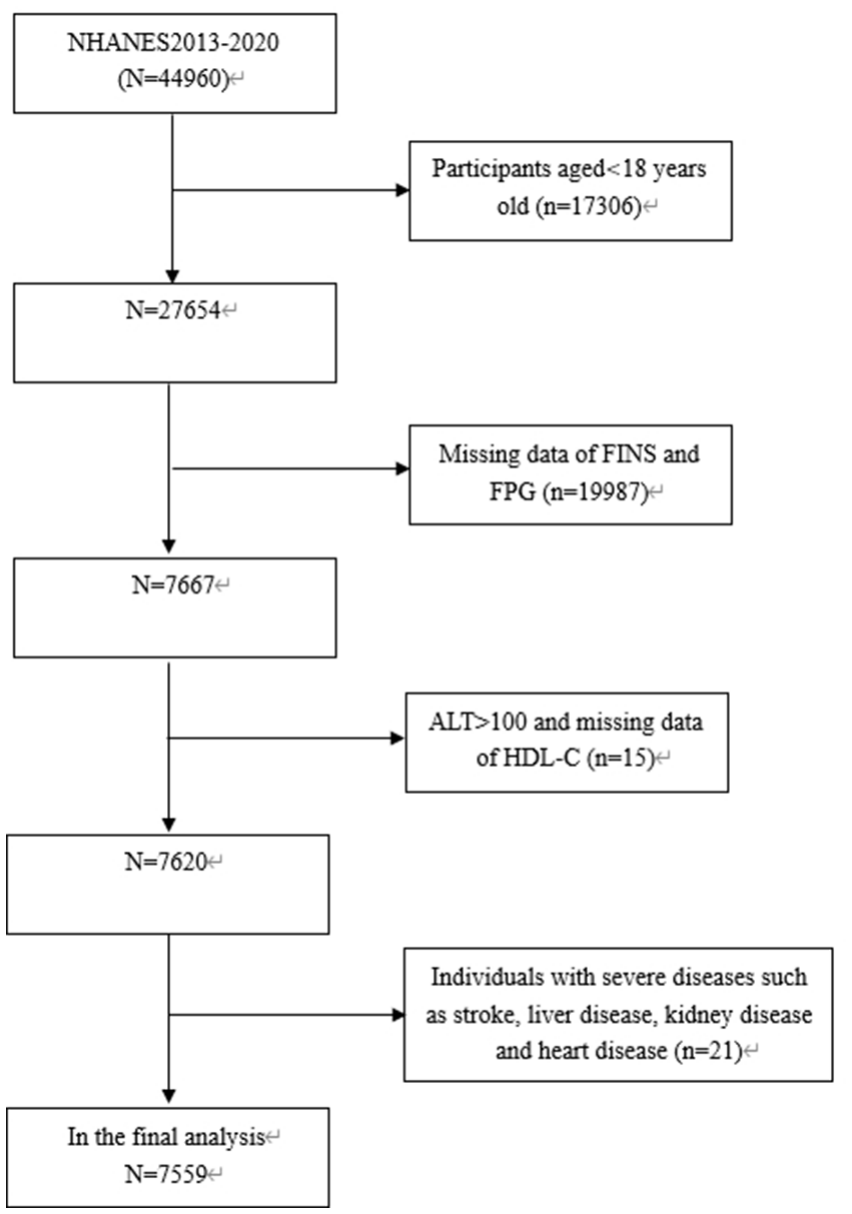
Flowchart of the sample selection from the 2013–2020 NHANES.

The study focused exclusively on subjects aged 18 years old and above (n = 27,654), among which, 20,055 subjects were eliminated: (1) Those with missing data on fasting insulin (FINS), ALT, HDL-C or FPG; (2) Those with ALT levels exceeding 100 IU/L, such as elevated levels are predominantly indicative of liver damage resulting from different forms of acute and chronic hepatitis; (3) Those with severe diseases such as stroke, heart disease, kidney disease and inflammatory disease. Consequently, the final analysis involved 7,599 subjects aged 18–80 years old (Figure 1).

The implementation of NHANES was granted approval by NCHS Ethics Review Board, and all subjects provided written informed consent (16).

### Anthropometric measurements

The following data were collected at admission, such as history of diabetes, alcohol intake, race, physical activity, education and physical measurements, including weight, waist circumference, height and blood pressure. Subjects who were obesity were defined as BMI ≥ 30 kg/m^2^, those with normal weight or overweight were defined as BMI <30 kg/m^2^.

TC, HbA1c, LDL-C, FINS, UA, FPG, triglycerides (TG), ALT, creatinine, AST, albumin and HDL-C were collected in blood samples. Less than 3% of values missed in total. Multiple imputation was performed for missing values. eGFR was estimated with the Modification of Diet in Renal Diseases (2). The detailed measuring method and acquisition process of each variable are available at www.cdc.gov/nchs/nhanes.

### Assessment of IR

IR was assessed through the HOMA-IR formula, and HOMA-IR was calculated as multiplied FPG (mmol/L) by FINS (IU/L) divided by 22.5 (2). IR was defined as HOMA-IR values equal to or greater than 2.69 (17, 18).

### Statistical analysis

It was worth noting that there were gender disparities in ALT, HDL, and ALT/HDL-C, and separate analyses were necessary for males and females. The assessment on normality for continuous variables involved expressing them as either median and interquartile range or mean ± standard deviation. In order to evaluate the differences between the two groups, t-test or Mann–Whitney U test was adopted for continuous variables, and chi-square tests were adopted for categorical variables. Furthermore, the association between ALT/HDL-C and metabolic risk factors was explored through Spearmen’s correlation. The subjects were divided into groups based on their ALT/HDL-C levels (≤12.94, 12.94–18.68, 18.68–16.08, ≥28.08 in the male group, ≤8.37, 8.37–11.43, 11.43–16.33, ≥16.33 in the female group). Variables demonstrating clinical significance and statistical significance in the univariate analysis (*p* < 0.05) were incorporated into the multivariate analyses. The association between ALT/HDL-C quartiles and the presence of IR was assessed with binary logistic regression models. In Model 1, no covariate was adjusted; In Model 2, adjustment was made for BMI and age. Based on Model 2, the race, moderate activities, diabetes, SBP, education level, WC, drinking, TC, HbA1c, DBP, serum albumin, eGFR, TG were added as covariates to Model 3. Subgroup analyses stratified by BMI (<30 kg/m2 and ≥ 30 kg/m2), gender (male and female), diabetes (yes and no), age (<50 and ≥ 50 years), moderate activities (yes and no), education level (high school or above and less than high school) and drinking (yes and no) were conducted (19–22). To examine the potential effect modification within subgroups, interaction terms were employed between subgroup indicators, followed by likelihood ratio tests. In order to identify potential nonlinear relationship between IR probabilities and ALT/HDL-C, generalized smooth curve fitting were adopted. ROC curve analyses were performed to evaluate the diagnostic efficacy of ALT/HDL-C in detecting IR. The statistical analysis was conducted with EmpowerStats software and R, with significance at *p* < 0.05.

## Results

### Characteristics of participants

As shown in Table 1, the prevalence of IR reached 46.0% in both genders. HOMA-IR, age, proportion individuals with diabetes, WC, BMI, SBP, DBP, HbA1c, FPG, FINS, TG, uric acid, ALT and ALT/HDL-C levels were all higher in IR subjects than those in non-IR subjects with both genders (*p* < 0.001). Furthermore, the proportion of moderate activities and HDL-C levels were lower in IR subjects than those in non-IR subjects with both genders.

**Table 1:** Baseline characteristics of the study population stratified by insulin resistance and gender.

	Male		*p*-value	Female		*P*-value
	IR positive	IR negative		IR positive	IR negative	
*N*	1968	2,313		2085	2,451	
Age, years	51.5 ± 17.9	47.3 ± 18.9	<0.001	50.3 ± 17.8	47.4 ± 18.6	<0.001
Race, %			<0.001			<0.001
Mexican American	16.9	12.7		20.2	10.9	
Other Hispanic	10.3	8.8		13.0	10.0	
Non-Hispanic white	38.4	39.1		31.5	39.9	
Non-Hispanic black	18.0	22.4		22.7	20.6	
Other race	16.5	17.0		12.6	18.7	
Moderate activities, %			0.009			<0.001
Yes	37.1	41.8		34.9	45.4	
No	62.9	58.2		65.1	54.6	
Diabetes			<0.001			<0.001
Yes	23.9	7.8		21.5	5.6	
No	76.1	92.2		78.5	94.4	
Education level			1.000			<0.001
Less than high school	23.4	23.4		25.7	17.4	
High school or above	76.6	76.6		74.3	82.6	
drinking, %			0.270			0.229
Current or ever	92.9	94.5		85.3	87.8	
Never	6.9	5.4		14.7	12.2	
Body mass index, Kg/m^2^	31.9 ± 6.7	25.8 ± 4.4	<0.001	34.0 ± 8.1	26.4 ± 6.0	<0.001
Waist circumference, cm	109.5 ± 15.6	93.0 ± 12.6	<0.001	107.9 ± 16.6	90.2 ± 13.7	<0.001
Systolic blood pressure, mmHg	127.7 ± 16.7	124.3 ± 18.5	<0.001	126.2 ± 18.8	119.5 ± 20.0	<0.001
Diastolic blood pressure, mmHg	72.3 ± 13.8	69.7 ± 12.8	<0.001	69.4 ± 12.7	67.5 ± 12.1	<0.001
Hemoglobin A1c, mmol/L	6.2 ± 1.4	5.5 ± 0.7	<0.001	6.2 ± 1.5	5.5 ± 0.6	<0.001
FPG, mmol/L	7.0 ± 2.7	5.7 ± 1.1	<0.001	6.8 ± 2.6	5.4 ± 0.8	<0.001
FINS, ng/ml	23.3 ± 24.3	6.2 ± 2.6	<0.001	22.6 ± 25.0	6.6 ± 2.5	<0.001
HOMA-IR	7.72 ± 9.11	1.55 ± 0.62	<0.001	7.15 ± 10.72	1.57 ± 0.60	<0.001
Albumin, g/dl	42.4 ± 3.5	43.2 ± 3.6	<0.001	40.2 ± 3.3	41.3 ± 3.5	<0.001
Creatinine, umol/L	89.8 ± 33.7	90.6 ± 55.9	0.573	67.9 ± 36.8	67.9 ± 40.3	0.989
eGFR, ml/min per 1.73m^2^	86.0 ± 25.3	87.8 ± 23.9	0.024	90.4 ± 28.6	90.3 ± 26.3	0.895
Uric acid, umol/L	379.1 ± 82.2	347.7 ± 74.2	<0.001	311.7 ± 81.1	273.4 ± 68.9	<0.001
ALT, IU/L	30.5 ± 15.2	23.3 ± 11.6	<0.001	21.7 ± 12.3	18.1 ± 9.3	<0.001
AST, IU/L	25.9 ± 10.5	24.6 ± 11.7	<0.001	22.2 ± 10.0	21.7 ± 11.4	0.119
Total cholesterol, mmol/L	4.81 ± 1.15	4.75 ± 1.03	0.094	4.95 ± 1.11	5.00 ± 1.09	0.198
Triglycerides, mmol/L	1.92 ± 1.58	1.20 ± 1.08	<0.001	1.64 ± 1.85	1.09 ± 0.66	<0.001
HDL-cholesterol, mmol/L	1.14 ± 0.29	1.39 ± 0.39	<0.001	1.36 ± 0.36	1.66 ± 0.44	<0.001
LDL-cholesterol, mmol/L	2.85 ± 0.94	2.82 ± 0.90	0.260	2.89 ± 0.92	2.83 ± 0.92	0.041
ALT/HDL-C	29.0 ± 18.6	18.3 ± 12.1	<0.001	17.3 ± 11.6	11.6 ± 7.1	<0.001

Values are mean ± SD or number (%). *P* < 0.05 was deemed significant (comparison between IR positive and IR negative). BMI, body mass index; FPG, fasting blood glucose; HbA1c, glycosylated hemoglobin; TC, total cholesterol; TG, triglyceride; HDL-c, High density lipoprotein cholesterol; LDL-c, Low density lipoprotein cholesterol.

### Correlation between clinical parameters and Alt/HDL-C

The correlation between metabolic parameters and ALT/HDL-C was analyzed with Spearman’s correlation and the results are shown in Table 2. The analysis revealed positive correlation between ALT/HDL-C and BMI, HbA1c, WC, TG, FPG, DBP, FINS, LDL-C, HOMA-IR in all subjects.

**Table 2:** Spearmen’s correlation of ALT/HDL levels with clinical and biochemical parameters.

Variable	Male		Female	
	*r*	*P*	*r*	*p*
BMI	0.404	<0.001	0.310	<0.001
WC	0.358	<0.001	0.328	<0.001
SBP	0.008	0.638	0.126	<0.001
DBP	0.147	<0.001	0.111	<0.001
HbA1c	0.073	<0.001	0.263	<0.001
TC	0.077	<0.001	−0.034	0.033
TG	0.462	<0.001	0.419	<0.001
LDL-C	0.135	<0.001	0.074	<0.001
FPG	0.162	<0.001	0.259	<0.001
FINS	0.449	<0.001	0.389	<0.001
HOMA-IR	0.445	<0.001	0.407	<0.001

### Correlation between IR and Alt/HDL-C

**Figure 2:**
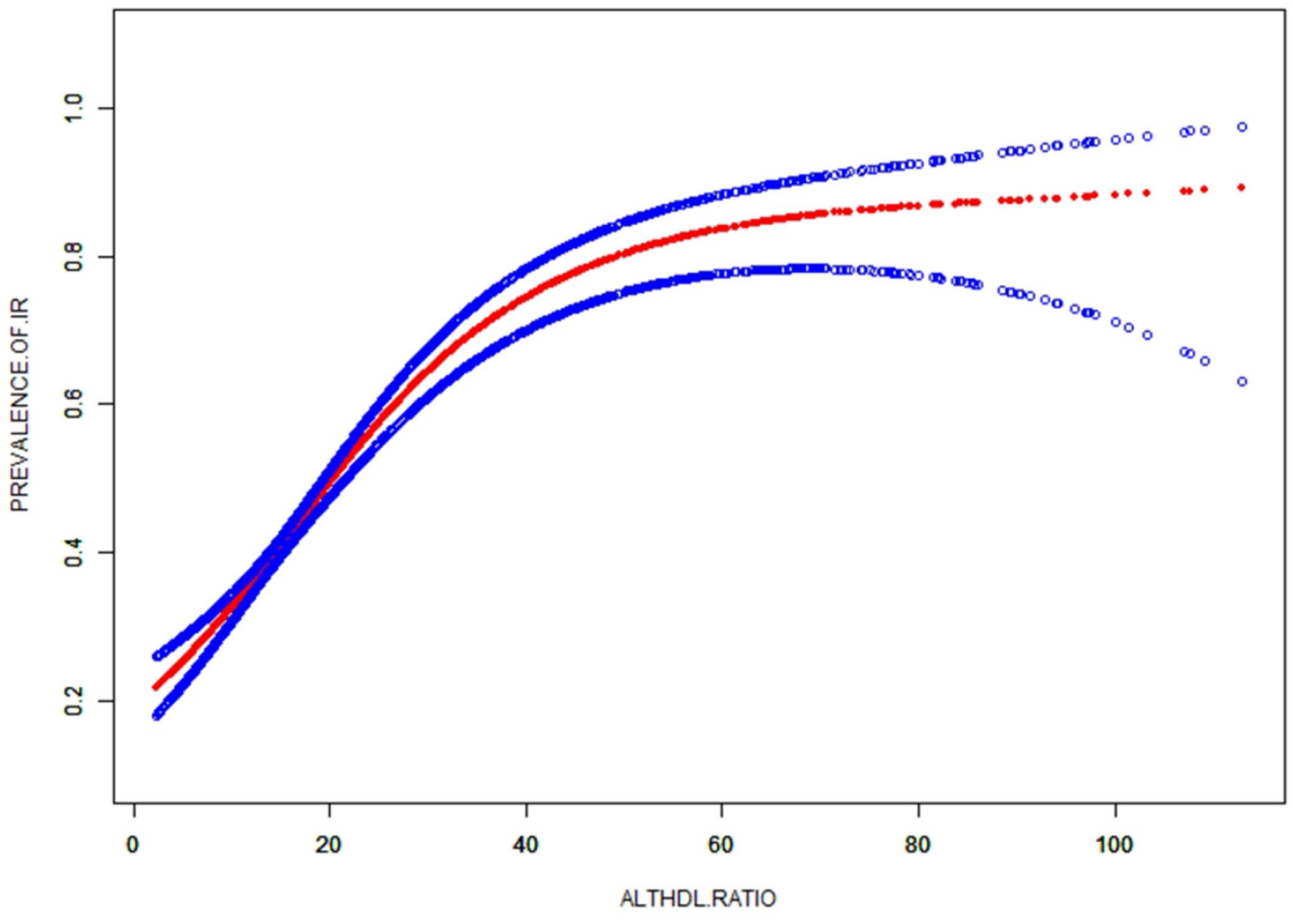
The smooth curve fit for the association between ALT/HDL-C and prevalence of IR.

Table 3 shows binary logistic analyses for the correlation between ALT/HDL-C quartiles with IR in subjects. In the unadjusted model, ALT/HDL-C was positively correlated with IR (OR = 1.06 in males and 1.09 in females). The association still existed in Model 2 (OR = 1.05 in males and 1.07 in females) and Model 3 (OR = 1.04 in both genders). In order to further investigate the association between IR and ALT/HDL-C, smooth curve fittings and generalized additive model were adopted (Table 4 and Figure 2). Among all subjects, the correlation between ALT/HDL-C and IR risk exhibited an inverted L-shaped curve, with inflection points at 33.62.

**Table 3:** Association of the insulin resistance with ALT/HDL-C quartiles.

	Crude model		Model I		Model II	
	OR (95% CI)	*P*	OR (95% CI)	*P*	OR (95% CI)	*P*
ALT/HDL-C	1.06 (1.05–1.06)	<0.001	1.05 (1.04–1.05)	<0.001	1.04 (1.03–1.05)	<0.001
Male						
ALT/HDL-C	1.06 (1.05, 1.07)	<0.001	1.05 (1.04, 1.05)	<0.001	1.04 (1.02–1.05)	<0.001
Q1	Ref		Ref		Ref	
Q2	1.95 (1.59–2.40)	<0.001	1.59 (1.26–2.02)	<0.001	1.70 (1.07–2.71)	0.025
Q3	4.12 (3.37–5.05)	<0.001	3.08 (2.44–3.89)	<0.001	3.44 (2.09–5.66)	<0.001
Q4	9.43 (7.62–11.68)	<0.001	6.32 (4.92–8.11)	<0.001	4.16 (2.39–7.23)	<0.001
Female						
ALT/HDL-C	1.09 (1.08., 1.10)	<0.001	1.07 (1.05, 1.08)	<0.001	1.04 (1.02–1.07)	<0.001
Q1	Ref		Ref		Ref	
Q2	1.60 (1.32–1.94)	0.008	1.37 (1.10–1.71)	0.005	1.11 (0.72–1.73)	0.628
Q3	3.00 (2.48–3.63)	<0.001	2.22 (1.79–2.75)	<0.001	1.58 (1.00–2.50)	0.048
Q4	6.25 (5.13–7.60)	<0.001	4.22 (3.39–5.26)	<0.001	2.02 (1.23–3.32)	0.006

Crude model: adjusted for none. Model I: adjusted for age and BMI. Model II: adjusted for gender (not adjusted for in the subgroup analyses), age, BMI, race, moderate activities, diabetes, education level, drinking, WC, SBP, DBP, HbA1c, eGFR, TC, TG, serum albumin.

**Table 4:** Threshold effect analysis of ALT/HDL-C on IR using the two-piecewise linear regression model.

UHR	Adjusted OR (95% CI) *p* value
Fitting by the standard linear model	1.049 (1.040, 1.059), <0.001
Fitting by the two-piecewise linear model	
Inflection point	33.62
ALT/HDL-C < 33.62	1.070 (1.058, 1.083), <0.001
AC > 33.62	1.018 (1.005, 1.032), 0.006
Log likelihood ratio	<0.001

### Subgroup analysis on the correlation between IR and Alt/HDL-C

**Figure 3:**
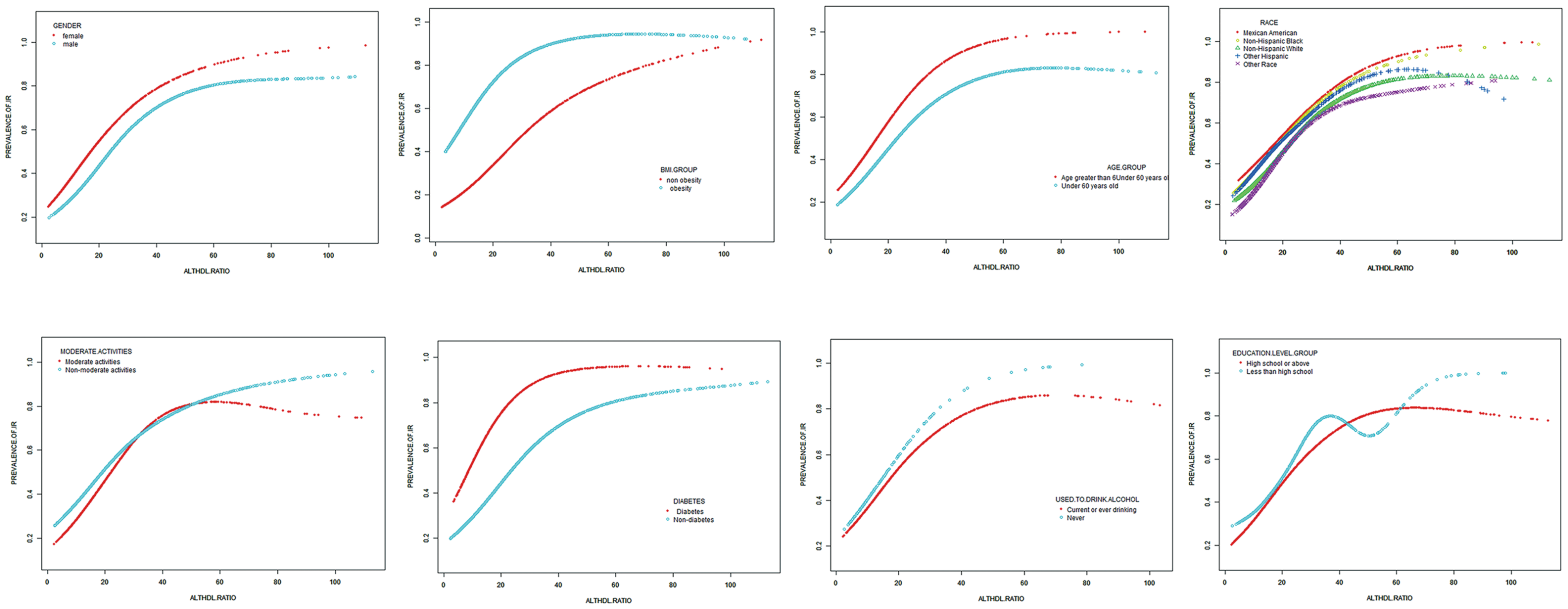
Subgroups analysis for the association between ALT/HDL-C and prevalence of IR by gender, age, race, BMI, diabetes, moderate activities, education level and drinking.

Subgroup analysis was conducted based on age, diabetes, sex, BMI, moderate activities and education level to investigate the association between ALT/HDL-C and the risk of IR in various commonly categorized populations. The findings indicate that there are significant statistical differences (*p* < 0.05) in the association between the ALT/HDL-C ratio and the risk of IR across various age and BMI groups. According to Table 5, the likelihood of developing IR exhibits notable disparities across distinct age groups, with individuals aged 50 and above displaying a heightened risk in comparison to those at the age < 50 (*p* = 0.003). In the subgroup analysis based on BMI, it was found that obese subjects have a higher risk of IR associated with the ALT/HDL-C ratio compared to non-obese subjects (OR: non-obese 1.03 *VS* obese 1.06; *p* = 0.044). Furthermore, upon employing smooth curve fittings to characterize the non-linear association, it was observed that the positive correlation between IR and ALT/HDL-C levels persisted in the majority of groups (Figure 3).

**Table 5:** Association between ALT/HDL and insulin resistance stratified by gender, age, race and BMI.

	OR (95%CI) p value	*P* for interaction
Stratified by gender		0.414
Male	1.04 (1.02–1.05), <0.001	
Female	1.04 (1.02–1.07), <0.001	
Stratified by race		0.840
Mexican American	1.07 (1.03, 1.11), <0.001	
Other Hispanic	1.06 (1.02, 1.11), 0.008	
Non-Hispanic white	1.04 (1.02, 1.07), <0.001	
Non-Hispanic black	1.03 (1.00, 1.06), 0.048	
Other race	1.03 (1.00, 1.05), <0.001	
Stratified by age		0.003
Age < 50 years old	1.03 (1.02, 1.04), <0.001	
Age ≥ 50 years old	1.08 (1.05, 1.11), <0.001	
Stratified by BMI		0.044
BMI < 30 kg/m^2^	1.03 (1.02, 1.06), <0.001	
BMI ≥ 30 kg/m^2^	1.06 (1.03, 1.09), <0.001	
Stratified by diabetes		0.159
Non-diabetes	1.03 (1.02, 1.05), <0.001	
Diabetes	1.05 (1.01, 1.09), 0.009	
Stratified by Moderate activities		0.109
No	1.03 (1.02, 1.05), <0.001	
Yes	1.05 (1.03, 1.07), <0.001	
Stratified by Education level		0.764
Less than high school	1.05 (1.02, 1.08), <0.001	
High school or above	1.04 (1.02, 1.05), <0.001	
Stratified by drinking		0.147
Current or ever drinking	1.04 (1.02, 1.05), <0.001	
Never	1.05 (1.00, 1.12), 0.046	

Gender, age, BMI, race, moderate activities, diabetes, education level (not adjusted for in the subgroup analyses), drinking, WC, SBP, DBP, HbA1c, TC, TG, eGFR and serum albumin were adjusted.

### Predictive value of Alt/HDL-C for IR

**Figure 4:**
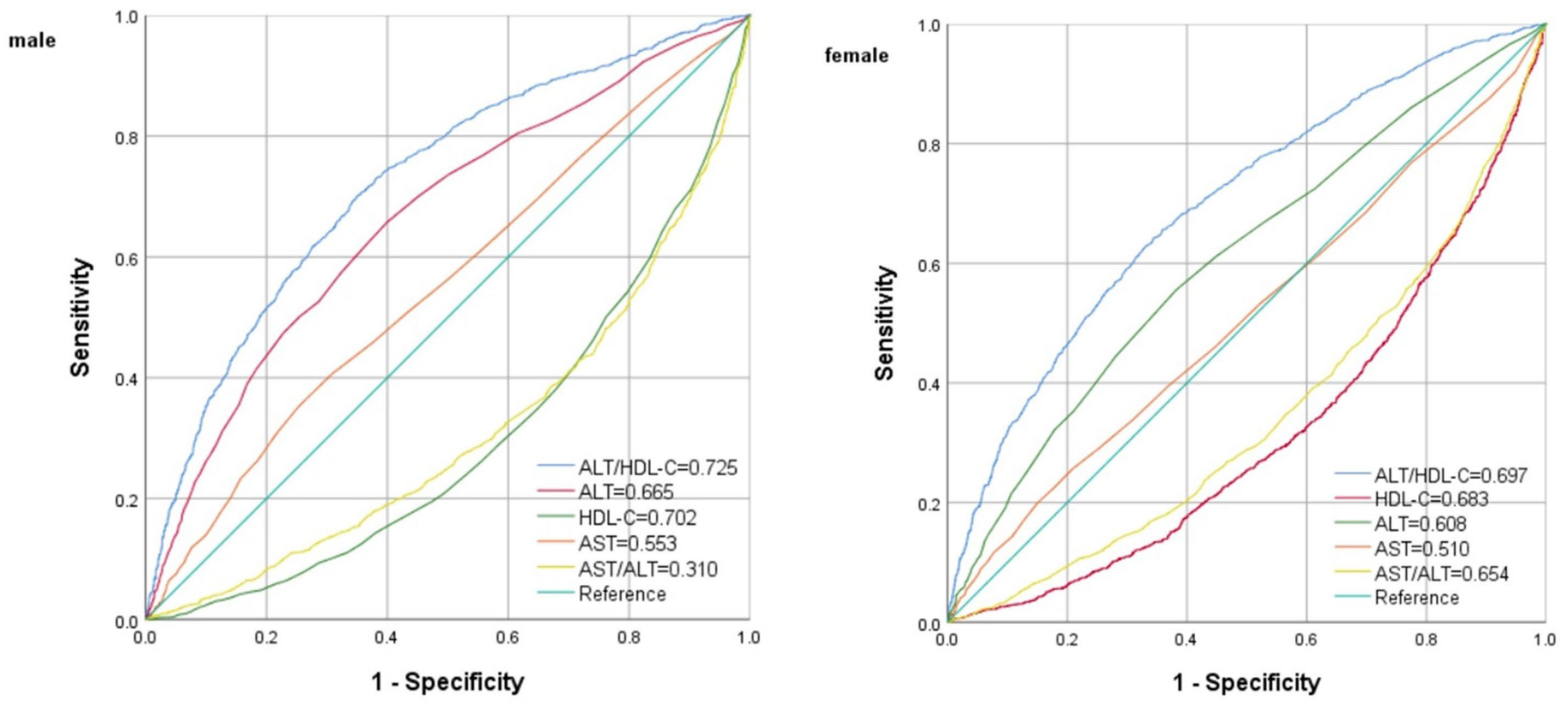
ROC analysis of ALT/HDL-C, ALT, HDL-C, AST and AST/ALT to IR among American adults.

The ROC curve in Figure 4 presents the diagnostic performance of ALT/HDL-C, HDL-C, ALT, AST and AST/ALT in identifying IR. Table 6 demonstrates that the AUC for ALT/HDL-C in the ROC analysis was 0.725 (95% CI: 0.709–0.742) for males and 0.696 (95% CI, 0.680–0.713) for females, exceeding ALT, HDL-C, AST and AST/ALT (*p* < 0.001), which suggests that ALT/HDL-C may serve as a superior indicator of IR compared with ALT or HDL-C alone, although its diagnostic accuracy remains somewhat limited.

**Table 6:** The results of ROC analysis of ALT/HDL, ALT, HDL-C, AST, AST/ALT and TG/HDL for the diagnosis of IR.

Nutritional indices	Cut-off	Sensitivity (%)	Specificity (%)	Youden’s index	AUC	95% CI
Male						
ALT/HDL-C	18.32	70.3	64.7	0.350	0.725	0.709–0.742
ALT	22.5	65.8	60.1	0.259	0.665	0.648–0.683
HDL-C	1.21	64.6	65.3	0.299	0.701	0.684–0.718
AST	26.5	35.4	74.6	0.100	0.553	0.534–0.572
AST/ALT	0.92	74.0	56.1	0.301	0.690	0.673–0.707
Female						
ALT/HDL-C	11.74	64.2	65.6	0.298	0.696	0.680–0.713
ALT	17.5	55.6	61.7	0.173	0.607	0.590–0.625
HDL-C	1.46	64.9	63.3	0.282	0.683	0.666–0.699
AST	26.5	20.0	85.0	0.05	0.510	0.491–0.528
AST/ALT	1.14	64.2	58.6	0.228	0.654	0.637–0.671

## Discussion

This study aims to investigate the connection between IR and ALT/HDL-C. Subsequently, stratified analyses were conducted to ascertain a notably stronger association between the two variables in subjects aged 50 and above, and those with obesity. Moreover, the ROC analysis demonstrated a substantial enhancement in the capacity to identify IR when utilizing the ALT/HDL-C ratio as compared to HDL-C, ALT, AST/ALT and AST. These findings indicate that ALT/HDL-C has the potential to be a valuable and straightforward biomarker for assessing the risk of IR.

The liver enzyme ALT is found to be strongly associated with hepatic fat accumulation and has also been linked to obesity and various components of MetS. Elevated ALT levels have been found to be associated with a higher prevalence of diabetes, MetS and cardiovascular diseases (23, 24). Additionally, ALT is considered as a predictive factor for non-alcoholic steatosis (25) and has been associated with both cardiovascular risk and IR in adolescents (26, 27). A reduction in HDL-C concentration is observed as a manifestation of MetS. Moreover, an elevation in HDL-C is widely recognized as a protective factor against IR (28). Furthermore, recent studies have proposed that the combination of HDL-C and ALT may serve as a more sensitive and novel biomarker for assessing inflammatory and metabolic disorders (14, 15).

As suggested in the study of Cao et al., the ALT/HDL-C composite index demonstrated enhanced predictive capabilities for diabetes compared to individual parameters (14). This amalgamation holds promise for potential applications in metabolic diseases. Based on the strong associations observed between the commonly employed atherosclerosis indicator HDL-C and the liver enzyme marker ALT with NAFLD (29–32). It is postulated that the amalgamation of ALT and HDL-C ratios is intricately associated with IR, potentially augmenting the capacity to detect IR.

The ALT/HDL-C ratio, the combination of the parameters of ALT and HDL-C, is predominantly investigated in clinical research (14). Conversely, establishing the threshold for ALT/HDL-C typically necessitates the accumulation of substantial clinical research evidence, thereby mitigating the selective disregard of chronic disease risks resulting from excessively high thresholds. In this study, the efficacy of various liver enzyme indicators was additionally compared in detecting IR. The findings demonstrate that ALT/HDL-C outperforms AST and AST/ALT significantly in identifying IR. The findings collectively indicate that the combination of HDL-C and ALT can potentially serve as a valuable tool for monitoring and diagnosing chronic diseases. The results obtained from the ROC analysis of this study suggest that an ALT/HDL-C (18.32 in males and 11.74 in females) can be considered as a screening threshold for identifying IR.

Furthermore, a more comprehensive subgroup analysis conducted in this study revealed intriguing findings, particularly highlighting a stronger association between IR and ALT/HDL-C in obese individuals and those aged 50 years and above. The main analysis of this study was presented as follows: Obese subjects generally exhibited elevated levels of ALT (33, 34) and decreased levels of HDL-C compared to non-obese subjects (35–38). A numerical analysis reveals that an increase in ALT or a decrease in HDL-C leads to an elevation in the ALT/HDL-C ratio. According to the findings, an elevated ALT/HDL-C ratio was indicative of an increased risk of IR in obese subjects. Additionally, advanced age and obesity were associated with poorer metabolic outcomes (39–41). Consequently, the heightened risk of IR observed in these populations in relation to ALT/HDL-C may be influenced by other metabolic pathways that contribute to these unfavorable outcomes.

Furthermore, an intriguing discovery of a previously unreported non-linear correlation between IR and ALT/HDL-C was made. It is likely that there is a saturating effect of IR risk when ALT/HDL-C reaches 33.62. The findings have the potential to provide new insights into the treatment and prevention of IR.

There are possible mechanistic explanations for the correlation between ALT/HDL-C and IR. It has been established that heightened levels of serum transaminases are linked to physical inactivity and obesity (42). Subjects with these characteristics exhibit lipid accumulation in hepatocytes and other tissues. ALT is recognized as the most reliable indicator of hepatic lipid accumulation (43). The elevated ALT can be interpreted as an indicator of subclinical systemic inflammation, with a correlation observed between ALT and proinflammatory molecules such as cytokines, CRP, and TNF-α (44). These molecules play a direct role in the pathogenesis of IR by modulating their signaling pathways (45). Additionally, ALT is primarily responsible for the conversion of alanine to pyruvate during hepatic glucose regulation, a process crucial for gluconeogenesis and closely linked to IR and the development of diabetes (46–48). HDL-C possesses various beneficial effects including reverse cholesterol transport, which can mitigate atherosclerosis, as well as anti-thrombotic, vasodilatory, anti-inflammatory and antiapoptotic properties (49). This study suggests that ALT/HDL-C, a combination of the lipid metabolism and inflammatory response, may serve as a potential indicator for IR.

The strengths of investigation encompass the extensive sample size and the nationwide representativeness of the United States. In addition, various confounding factors, including diabetes, age, drinking status, gender, physical activity and BMI, have been taken into account. Nevertheless, there are certain constraints in this study. First of all, the cross-sectional studies do not allow us to establish a cause-and-effect association between ALT/HDL-C and IR. Secondly, HOMA-IR was adopted as a means to assess IR. Although it is not taken as a gold standard, it is widely used because of its practicality. Thirdly, the AUC for ALT/HDL-C in the ROC analysis was close to 0.7, indicating limited diagnostic ability. Fourthly, it is crucial to verify the connection between ALT/HDL-C and IR in diverse populations and ethnicities, as this study solely focuses on American.

## Conclusion

In conclusion, findings from this investigation conducted on American adults reveal a positive correlation between IR and ALT/HDL-C. This association is particularly significant among individuals with obesity and those aged 50 or older. It may be an effective indicator to identify IR in American and prevent disease progression.

## Data availability statement

Publicly available datasets were analyzed in this study. This data can be found at: NHANES, www.cdc.gov/nchs/NHANEs/.

## Ethics statement

The studies involving humans were approved by National Center for Health Statistics Ethics Review Board. The studies were conducted in accordance with the local legislation and institutional requirements. The participants provided their written informed consent to participate in this study.

## Author contributions

XZ: Writing – original draft, Conceptualization. JX: Conceptualization, Data curation, Investigation, Writing – original draft. HD: Writing – review & editing, Writing – original draft.

## References

[1] Tam CS Xie W Johnson WD Cefalu WT Redman LM Ravussin E. Defining insulin resistance from hyperinsulinemic-euglycemic clamps. Diabetes Care. (2012) 35:1605–10. doi: 10.2337/dc11-2339, PMID: 22511259 PMC3379600

[2] Wallace TM Levy JC Matthews DR. Use and abuse of HOMA modeling. Diabetes Care. (2004) 27:1487–95. doi: 10.2337/diacare.27.6.1487 15161807

[3] Espinel-Bermudez MC Robles-Cervantes JA Del Sagrario Villarreal-Hernandez L Villasenor-Romero JP Hernandez-Gonzalez SO Gonzalez-Ortiz M. Insulin resistance in adult primary care patients with a surrogate index, Guadalajara, Mexico, 2012. J Investig Med. (2015) 63:247–50. doi: 10.1097/JIM.0000000000000130, PMID: 25503090 PMC4304577

[4] Borai A Livingstone C Ferns GA. The biochemical assessment of insulin resistance. Ann Clin Biochem. (2007) 44:324–42. doi: 10.1258/000456307780945778 17594780

[5] Rudvik A Mansson M. Evaluation of surrogate measures of insulin sensitivity - correlation with gold standard is not enough. BMC Med Res Methodol. (2018) 18:64. doi: 10.1186/s12874-018-0521-y, PMID: 29940866 PMC6019831

[6] Burke JP Hale DE Hazuda HP Stern MP. A quantitative scale of acanthosis nigricans. Diabetes Care. (1999) 22:1655–9. doi: 10.2337/diacare.22.10.1655, PMID: 10526730

[7] Sung KC Jeong WS Wild SH Byrne CD. Combined influence of insulin resistance, overweight/obesity, and fatty liver as risk factors for type 2 diabetes. Diabetes Care. (2012) 35:717–22. doi: 10.2337/dc11-1853, PMID: 22338098 PMC3308286

[8] Wu CZ Hsieh CH Lu CH Pei D Chen JS Chen YL. First-phase insulin secretion is positively correlated with alanine aminotransferase in young adults. Adv Clin Exp Med. (2021) 30:35–40. doi: 10.17219/acem/128229, PMID: 33529505

[9] Leavens KF Birnbaum MJ. Insulin signaling to hepatic lipid metabolism in health and disease. Crit Rev Biochem Mol Biol. (2011) 46:200–15. doi: 10.3109/10409238.2011.562481 21599535

[10] von Eckardstein A Sibler RA. Possible contributions of lipoproteins and cholesterol to the pathogenesis of diabetes mellitus type 2. Curr Opin Lipidol. (2011) 22:26–32. doi: 10.1097/MOL.0b013e3283412279, PMID: 21102330

[11] Han T Cheng Y Tian S Wang L Liang X Duan W. Changes in triglycerides and high-density lipoprotein cholesterol may precede peripheral insulin resistance, with 2-h insulin partially mediating this unidirectional relationship: a prospective cohort study. Cardiovasc Diabetol. (2016) 15:154. doi: 10.1186/s12933-016-0469-3, PMID: 27814764 PMC5095985

[12] Rachek LI. Free fatty acids and skeletal muscle insulin resistance. Prog Mol Biol Transl Sci. (2014) 121:267–92. doi: 10.1016/B978-0-12-800101-1.00008-9 24373240

[13] Karhapaa P Malkki M Laakso M. Isolated low HDL cholesterol. An insulin-resistant state. Diabetes. (1994) 43:411–7. PMID: 8314013 10.2337/diab.43.3.411

[14] Cao C Hu H Han Y Yuan S Zheng X Zhang X. The nonlinear correlation between alanine aminotransferase to high-density lipoprotein cholesterol ratio and the risk of diabetes: a historical Japanese cohort study. BMC Endocr Disord. (2023) 23:124. doi: 10.1186/s12902-023-01382-7, PMID: 37248447 PMC10226242

[15] He S Yu C Kuang M Qiu J Yang R Zhang S. Alanine aminotransferase to high-density lipoprotein cholesterol ratio is positively correlated with the occurrence of diabetes in the Chinese population: a population-based cohort study. Front Endocrinol (Lausanne). (2023) 14:1266692. doi: 10.3389/fendo.2023.1266692, PMID: 38089616 PMC10715265

[16] Zipf G Chiappa M Porter KS Ostchega Y Lewis BG Dostal J. National health and nutrition examination survey: plan and operations, 1999-2010. Vital Health Stat. (2013) 1:1–37. 25078429

[17] Zhou X Xu J. Association between serum uric acid-to-high-density lipoprotein cholesterol ratio and insulin resistance in patients with type 2 diabetes mellitus. J Diabetes Investig. (2023) 15:113–20. doi: 10.1111/jdi.14086 PMC10759725 37737515

[18] Zhou M Zhu L Cui X Feng L Zhao X He S. The triglyceride to high-density lipoprotein cholesterol (TG/HDL-C) ratio as a predictor of insulin resistance but not of beta cell function in a Chinese population with different glucose tolerance status. Lipids Health Dis. (2016) 15:104. doi: 10.1186/s12944-016-0270-z, PMID: 27267043 PMC4895977

[19] Zeng P Cai X Yu X Gong L. Markers of insulin resistance associated with non-alcoholic fatty liver disease in non-diabetic population. Sci Rep. (2023) 13:20470. doi: 10.1038/s41598-023-47269-4, PMID: 37993481 PMC10665395

[20] Di Murro E Di Giuseppe G Soldovieri L Moffa S Improta I Capece U. Physical activity and type 2 diabetes: in search of a personalized approach to improving beta-cell function. Nutrients. (2023) 15:202. doi: 10.3390/nu15194202, PMID: 37836486 PMC10574038

[21] Ortega FB Ruiz JR Hurtig-Wennlof A Meirhaeghe A Gonzalez-Gross M Moreno LA. Physical activity attenuates the effect of low birth weight on insulin resistance in adolescents: findings from two observational studies. Diabetes. (2011) 60:2295–9. doi: 10.2337/db10-1670, PMID: 21752955 PMC3161315

[22] Arjmand B Ebrahimi Fana S Ghasemi E Kazemi A Ghodssi-Ghassemabadi R Dehghanbanadaki H. Metabolic signatures of insulin resistance in non-diabetic individuals. BMC Endocr Disord. (2022) 22:212. doi: 10.1186/s12902-022-01130-3, PMID: 36002887 PMC9404631

[23] Hartman C Rennert HS Rennert G Elenberg Y Zuckerman E. Prevalence of elevated liver enzymes and comorbidities in children and adolescents with overweight and obesity. Acta Paediatr. (2021) 110:985–92. doi: 10.1111/apa.15469, PMID: 32649794

[24] Porter SA Pedley A Massaro JM Vasan RS Hoffmann U Fox CS. Aminotransferase levels are associated with cardiometabolic risk above and beyond visceral fat and insulin resistance: the Framingham heart study. Arterioscler Thromb Vasc Biol. (2013) 33:139–46. doi: 10.1161/ATVBAHA.112.300075, PMID: 23162012 PMC3593729

[25] Vos MB Abrams SH Barlow SE Caprio S Daniels SR Kohli R. NASPGHAN clinical practice guideline for the diagnosis and treatment of nonalcoholic fatty liver disease in children: recommendations from the expert committee on NAFLD (ECON) and the north American Society of Pediatric Gastroenterology, hepatology and nutrition (NASPGHAN). J Pediatr Gastroenterol Nutr. (2017) 64:319–34. doi: 10.1097/MPG.0000000000001482, PMID: 28107283 PMC5413933

[26] Klein M Iazzettii L Speiser P Carey D Shelov S Accacha S. Alanine transferase: an independent indicator of adiposity related comorbidity risk in youth. J Diabetes. (2015) 7:649–56. doi: 10.1111/1753-0407.12221, PMID: 25266069

[27] Patel DA Srinivasan SR Chen W Berenson GS. Serum alanine aminotransferase and its association with metabolic syndrome in children: the Bogalusa heart study. Metab Syndr Relat Disord. (2011) 9:211–6. doi: 10.1089/met.2010.0086, PMID: 21476865 PMC3125570

[28] Anan F Yonemochi H Masaki T Takahashi N Fukunaga N Teshima Y. High-density lipoprotein cholesterol and insulin resistance are independent and additive markers of left ventricular hypertrophy in essential hypertension. Hypertens Res. (2007) 30:125–31. doi: 10.1291/hypres.30.125, PMID: 17460382

[29] Chen ZW Chen LY Dai HL Chen JH Fang LZ. Relationship between alanine aminotransferase levels and metabolic syndrome in nonalcoholic fatty liver disease. J Zhejiang Univ Sci B. (2008) 9:616–22. doi: 10.1631/jzus.B0720016, PMID: 18763311 PMC2491691

[30] Schindhelm RK Diamant M Dekker JM Tushuizen ME Teerlink T Heine RJ. Alanine aminotransferase as a marker of non-alcoholic fatty liver disease in relation to type 2 diabetes mellitus and cardiovascular disease. Diabetes Metab Res Rev. (2006) 22:437–43. doi: 10.1002/dmrr.666, PMID: 16832839

[31] Omagari K Takamura R Matsutake S Ichimura M Kato S Morikawa S. Serum alanine aminotransferase concentration as a predictive factor for the development or regression of fatty liver. J Clin Biochem Nutr. (2011) 49:200–6. doi: 10.3164/jcbn.11-27, PMID: 22128220 PMC3208017

[32] Chang Y Ryu S Sung E Jang Y. Higher concentrations of alanine aminotransferase within the reference interval predict nonalcoholic fatty liver disease. Clin Chem. (2007) 53:686–92. doi: 10.1373/clinchem.2006.081257, PMID: 17272484

[33] Samadi N Cembrowski GS Chan J. Effect of waist circumference on reference intervals of liver-related enzyme tests in apparently healthy adult Mexican Americans, black and white Americans. Clin Biochem. (2007) 40:206–12. doi: 10.1016/j.clinbiochem.2006.11.007 17222815

[34] Bekkelund SI Jorde R. Alanine aminotransferase and body composition in obese men and women. Dis Markers. (2019) 2019:1695874. doi: 10.1155/2019/1695874 31534560 PMC6732629

[35] Davis CE Williams DH Oganov RG Tao SC Rywik SL Stein Y. Sex difference in high density lipoprotein cholesterol in six countries. Am J Epidemiol. (1996) 143:1100–6. doi: 10.1093/oxfordjournals.aje.a008686, PMID: 8633598

[36] Kauma H Savolainen MJ Heikkila R Rantala AO Lilja M Reunanen A. Sex difference in the regulation of plasma high density lipoprotein cholesterol by genetic and environmental factors. Hum Genet. (1996) 97:156–62. doi: 10.1007/BF02265258, PMID: 8566946

[37] Mooradian AD Albert SG Haas MJ. Low serum high-density lipoprotein cholesterol in obese subjects with normal serum triglycerides: the role of insulin resistance and inflammatory cytokines. Diabetes Obes Metab. (2007) 9:441–3. doi: 10.1111/j.1463-1326.2006.00636.x, PMID: 17391174

[38] Anagnostis P Stevenson JC Crook D Johnston DG Godsland IF. Effects of menopause, gender and age on lipids and high-density lipoprotein cholesterol subfractions. Maturitas. (2015) 81:62–8. doi: 10.1016/j.maturitas.2015.02.262, PMID: 25804951

[39] Nannini DR Joyce BT Zheng Y Gao T Liu L Yoon G. Epigenetic age acceleration and metabolic syndrome in the coronary artery risk development in young adults study. Clin Epigenetics. (2019) 11:160. doi: 10.1186/s13148-019-0767-1, PMID: 31730017 PMC6858654

[40] Pucci G Alcidi R Tap L Battista F Mattace-Raso F Schillaci G. Sex-and gender-related prevalence, cardiovascular risk and therapeutic approach in metabolic syndrome: a review of the literature. Pharmacol Res. (2017) 120:34–42. doi: 10.1016/j.phrs.2017.03.008 28300617

[41] Ramirez-Manent JI Jover AM Martinez CS Tomas-Gil P Marti-Lliteras P Lopez-Gonzalez AA. Waist circumference is an essential factor in predicting insulin resistance and early detection of metabolic syndrome in adults. Nutrients. (2023) 15:257. doi: 10.3390/nu15020257, PMID: 36678129 PMC9861022

[42] Villegas R Xiang YB Elasy T Cai Q Xu W Li H. Liver enzymes, type 2 diabetes, and metabolic syndrome in middle-aged, urban Chinese men. Metab Syndr Relat Disord. (2011) 9:305–11. doi: 10.1089/met.2011.0016, PMID: 21495862 PMC3142636

[43] Mohamed J Nazratun Nafizah AH Zariyantey AH Budin SB. Mechanisms of diabetes-induced liver damage: the role of oxidative stress and inflammation. Sultan Qaboos Univ Med J. (2016) 16:e132–41. doi: 10.18295/squmj.2016.16.02.002, PMID: 27226903 PMC4868511

[44] Wang CS Chang TT Yao WJ Wang ST Chou P. Impact of increasing alanine aminotransferase levels within normal range on incident diabetes. J Formos Med Assoc. (2012) 111:201–8. doi: 10.1016/j.jfma.2011.04.004, PMID: 22526208

[45] Chen L Chen R Wang H Liang F. Mechanisms linking inflammation to insulin resistance. Int J Endocrinol. (2015) 2015:508409. doi: 10.1155/2015/508409 26136779 PMC4468292

[46] Hanley AJ Wagenknecht LE Festa A D'Agostino RB Jr Haffner SM. Alanine aminotransferase and directly measured insulin sensitivity in a multiethnic cohort: the insulin resistance atherosclerosis study. Diabetes Care. (2007) 30:1819–27. doi: 10.2337/dc07-0086, PMID: 17429060

[47] Qian K Zhong S Xie K Yu D Yang R Gong DW. Hepatic ALT isoenzymes are elevated in gluconeogenic conditions including diabetes and suppressed by insulin at the protein level. Diabetes Metab Res Rev. (2015) 31:562–71. doi: 10.1002/dmrr.2655, PMID: 25865565 PMC4696510

[48] Ballestri S Zona S Targher G Romagnoli D Baldelli E Nascimbeni F. Nonalcoholic fatty liver disease is associated with an almost twofold increased risk of incident type 2 diabetes and metabolic syndrome. Evidence from a systematic review and meta-analysis. J Gastroenterol Hepatol. (2016) 31:936–44. doi: 10.1111/jgh.13264 26667191

[49] Nagao M Nakajima H Toh R Hirata KI Ishida T. Cardioprotective effects of high-density lipoprotein beyond its anti-atherogenic action. J Atheroscler Thromb. (2018) 25:985–93. doi: 10.5551/jat.RV17025, PMID: 30146614 PMC6193192

